# What's in a name? Upper extremity fracture eponyms (Part 1)

**DOI:** 10.1186/s12245-015-0075-2

**Published:** 2015-07-30

**Authors:** Philip Kin-Wai Wong, Tarek N. Hanna, Waqas Shuaib, Stephen M. Sanders, Faisal Khosa

**Affiliations:** Department of Radiology and Imaging Sciences, Emory University School of Medicine, 1364 Clifton Road, Atlanta, GA 30322 USA; Division of Emergency Radiology, Department of Radiology and Imaging Sciences, Emory University Hospital Midtown, 550 Peachtree Street NE, Atlanta, GA 30308 USA; Department of Radiology and Imaging Sciences, Emory University Hospital Midtown, 550 Peachtree Street NE, Atlanta, GA 30308 USA; Department of Emergency Medicine, Emory University Hospital, 531 Asbury Circle, Annex Building, Suite N340, Atlanta, GA 30322 USA

**Keywords:** Eponyms, Fractures, Upper extremities, Radiology

## Abstract

Eponymous extremity fractures are commonly encountered in the emergency setting. Correct eponym usage allows rapid, succinct communication of complex injuries. We will review both common and less frequently encountered extremity fracture eponyms, focusing on imaging features to identify and differentiate these injuries. We focus on plain radiographic findings, with supporting computed tomography (CT) images. For each injury, important radiologic descriptors are discussed which may need to be communicated to consultants. Aspects of management and follow-up imaging recommendations are included. This is a two-part review: Part 1 focuses on fracture eponyms of the upper extremity, while Part 2 covers fracture eponyms of the lower extremity.

## Introduction

Eponyms are embedded throughout medicine; they can be found in medical literature, textbooks, and even mass media. Their use allows physicians to quickly provide a concise description of a complex injury pattern. Eponymous extremity fractures are commonly encountered in the emergency setting and are frequently used in interactions amongst radiologists, emergency clinicians, and orthopedists. Unfortunately, the imprecise use of eponyms can result in confusion and miscommunication [1]. In this two-part series, our goal is to provide emergency providers with consistent, accurate definitions and depictions of commonly and less frequently encountered extremity fracture eponyms, keying in on important imaging features that differentiate these fractures. We illustrate fundamental descriptors of each injury that a clinician should expect in a radiology report. We also briefly review the mechanism of each injury, associated complications, any follow-up imaging needed, and treatment.

## Review: Upper extremity fracture eponyms

### Hill-Sachs impaction fracture

**Fig. 1:**
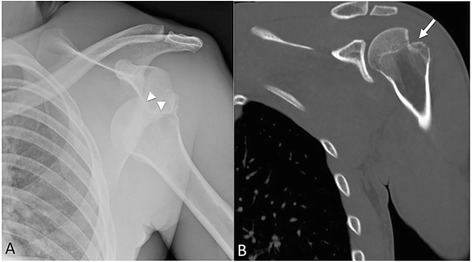
Hill-Sachs impaction fracture. **a** AP left shoulder radiograph, with anterior inferior humeral head dislocation. Internal rotation positioning. The superolateral humeral head is impacted and lodged on the inferior osseous glenoid (*arrowheads*). **b** Coronal CT confirmed pronounced Hill-Sachs fracture (*arrow*). Although more substantial than commonly seen, this Hill-Sachs illustrates the mechanism and imaging findings. Subtle Hill-Sachs fractures may be a subtle trough, rather than angular fracture seen here

Originally reported by radiologists Arthur Hill and David Sachs, the Hill-Sachs deformity is an impaction fracture of the posterolateral aspect of the humeral head found almost exclusively in anterior shoulder dislocations (Fig. 1). Depending on its location and severity, this defect may contribute to anterior shoulder instability and predispose to recurrent dislocations [2]. On conventional radiographs, this fracture is optimally detected on an anterior-posterior (AP) internally rotated view of the shoulder [3]. Computed tomography (CT) best depicts the location and size of Hill-Sachs lesions and provides concurrent evaluation for other commonly associated glenohumeral joint injuries such as the bony Bankart fracture [2]. Treatment for recurrent dislocations and shoulder instability is surgical [2].

### Bankart fracture

**Fig. 2:**
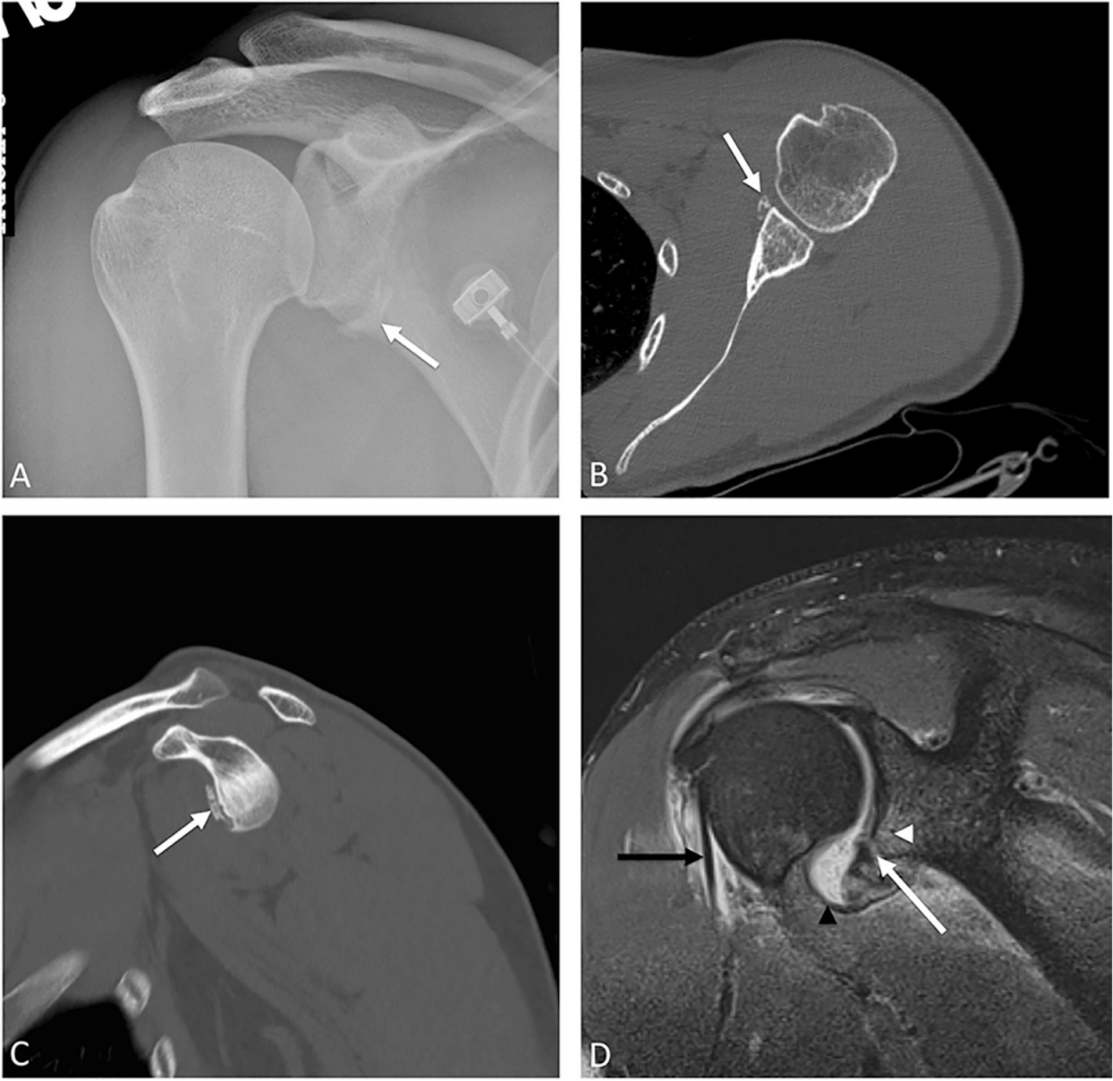
Bony Bankart on radiograph (**a**), CT (**b**, **c**), and MRI (**d**) in several patients. **a** Crescentic bone fragment at the inferomedial aspect of the glenohumeral (GH) joint space (*white arrow*) in this young man who was post anterior dislocation and reduction. This is a medially displaced bony Bankart fracture. Axial (**b**) and sagittal CT (**c**) with typical appearance of anterior glenoid fracture (*white arrows*). **d** Oblique coronal proton density fat-saturated MR shows inferior glenoid marrow edema (*arrowhead*) and bony Bankart fracture (*arrow*). Joint effusion is present, with distention of the inferior GH capsule (*black arrowhead*). Long head biceps tendon is surrounded by fluid (*black arrow*), which tracks in the biceps sheath—and extension of the articular space

Named after the renowned English orthopedic surgeon Arthur Bankart, the Bankart lesion is often associated with the Hill-Sachs lesion due to their common mechanism of injury. The soft tissue Bankart lesion is an injury to the anterior or anteroinferior glenoid labrum, the fibrocartilagenous structure that surrounds and deepens the bony glenoid. An osseous or bony Bankart fracture is a chip fracture of the anterior inferior glenoid cortical rim on which the labrum rests (Fig. 2). The inferior glenoid rim should be closely interrogated on pre- and post-reduction shoulder radiographs. Any concern for this injury warrants non-emergent magnetic resonance imaging (MRI), which provides evaluation for labral as well as osseous injury [4]. Similar to Hill-Sachs, this lesion may result in anterior shoulder joint instability and recurrent dislocations. These lesions can be diagnosed with a CT (especially 3-D reconstructions) or MRI. Surgical techniques to address recurrent anterior instability include labral repair or bone reconstruction if the degree of bone loss is severe [4].

### Holstein-Lewis fracture

The Holstein-Lewis fracture was first described by American orthopedic surgeons Arthur Holstein and Gwilym Lewis [5]. The injury is a spiral fracture of the distal third of the humerus, typically as a consequence of blunt trauma, with the distal fragment displaced such that the proximal aspect is deviated radially. At this particular level, the radial nerve courses distally through the lateral intermuscular septum and is in direct contact with the adjacent bone, leaving the nerve susceptible to injury [5]. The Holstein-Lewis fracture thus has an association with radial nerve palsy (22 %) [6]. Lateral and AP radiographs are typically sufficient for imaging diagnosis. Recognizing this fracture pattern should alert the provider to the possibility of radial nerve injury and prompt a more extensive neurologic assessment. Debate still exists on whether Holstein-Lewis fractures should be managed conservatively versus early open reduction internal fixation with radial nerve exploration as traumatic injury to the radial nerve has been shown to heal spontaneously [6, 7].

### Galeazzi fracture-dislocation (Piedmont fracture/reverse Monteggia)

**Fig. 3:**
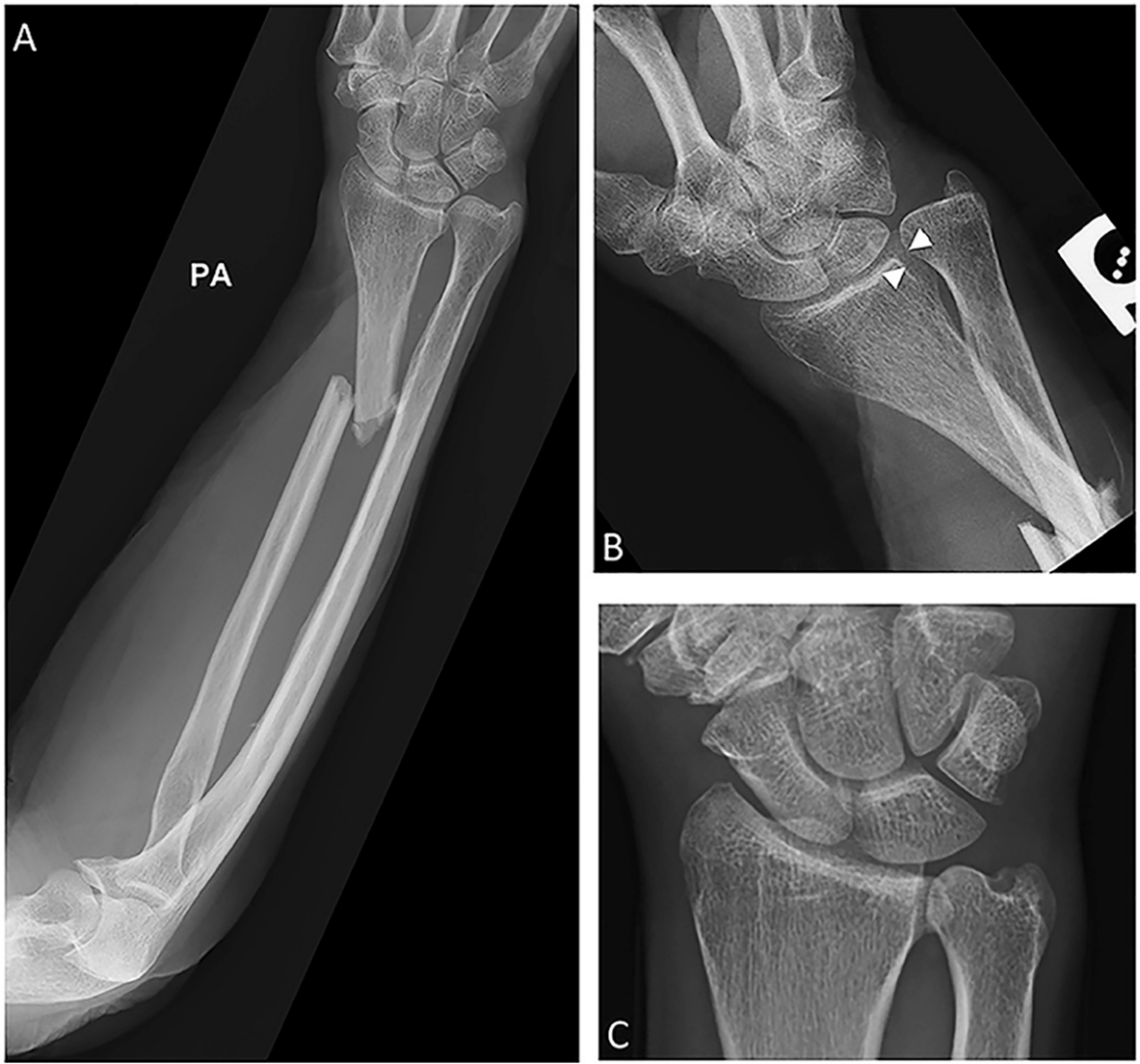
Galeazzi fracture. **a** PA forearm radiograph with displaced fracture of the distal one third of the radial shaft. **b** Wrist radiograph in the same patient demonstrates subluxation of the distal radioulnar joint (DRUJ), with mild DRUJ widening, measuring 5 mm (*arrowheads*), and mild radial foreshortening. **c** Comparison normal wrist radiograph. Notice the small caliber of a normal, tight DRUJ

Named after Italian surgeon Ricardo Galeazzi, the Galeazzi fracture-dislocation is a fracture of the middle to distal third of the radius associated with dislocation or disruption of the distal radioulnar joint (DRUJ) (Fig. 3). It has been previously termed the “fracture of necessity” in reference to the frequent need for surgical intervention [8]. The mechanism of injury is a fall on an outstretched pronated hand. Recognition of subluxation or dislocation of the DRUJ is paramount. Radiographic findings such as widening of the DRUJ on AP radiograph, a distance of 5 mm or more between the distal radius and ulna on the lateral radiograph, dislocation of the ulna on lateral view, and greater than 5 mm of radial shortening or impaction are all suggestive of DRUJ injury [4, 9–11]. A fracture of the radial shaft in children may be associated with separation of the distal ulnar epiphysis without disruption of the DRUJ. In adults, a fracture of the radial shaft may be associated with a fracture of the distal ulna. These entities are known as Galeazzi equivalent and should be treated similarly [12]. In adults, open reduction and internal fixation with plate and screw fixation is the preferred method of treatment. This also allows for intraoperative assessment of DRUJ stability and ligamentous repair if necessary [8, 12]. The Galeazzi fracture may sometimes be referred to as the Piedmont fracture or the reverse Monteggia fracture [13].

### Monteggia fracture-dislocation

**Fig. 4:**
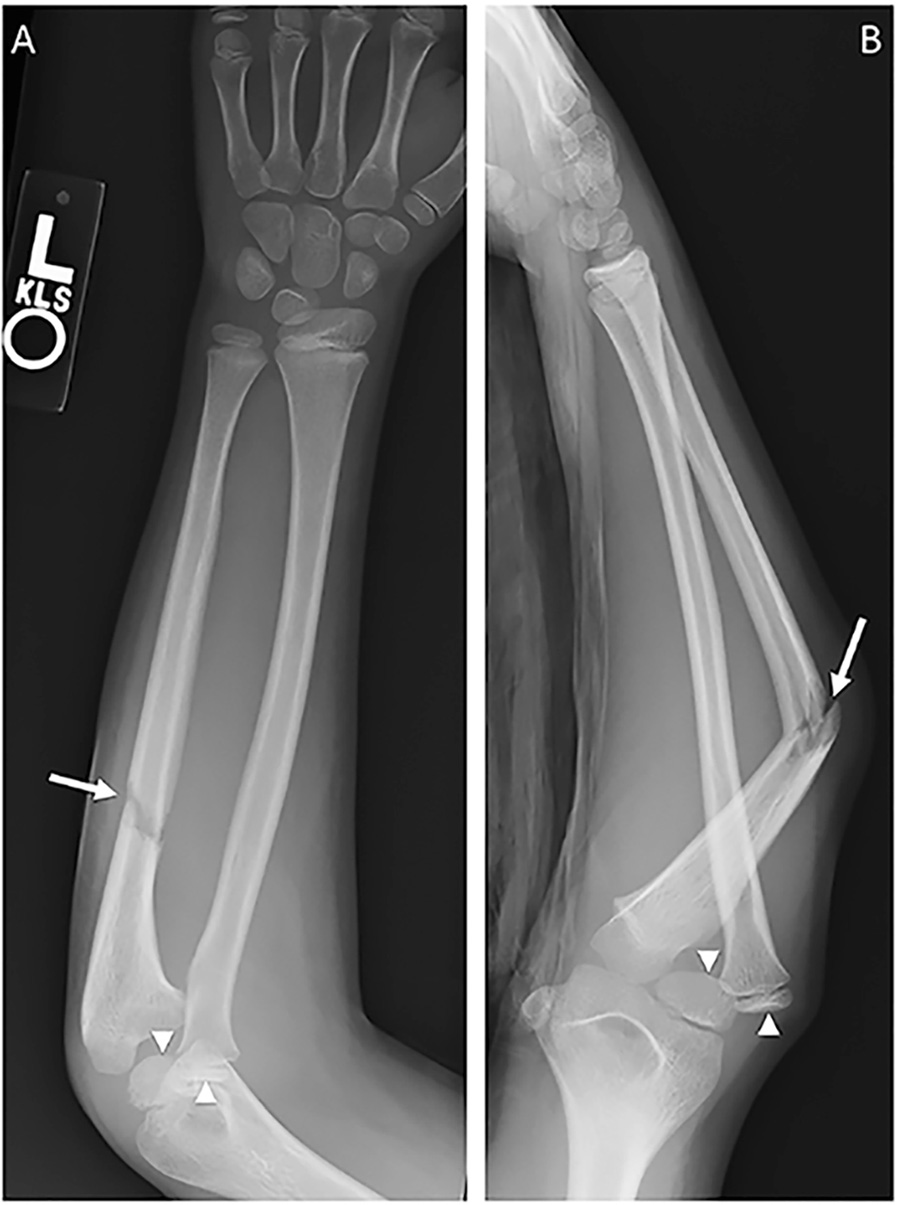
Monteggia fracture. PA (**a**) and lateral (**b**) radiographs of the forearm demonstrate angulated fracture of the proximal ulna (*arrow*) and dislocation of the radial head from the capitellum (*arrowheads* on both the radial head and capitellum)

The Monteggia fracture-dislocation, first described in 1814 by the Milanese surgeon Giovanni Battista Monteggia, refers to the fracture of the proximal ulna with dislocation of the proximal radioulnar and radiocapitellar joints (Fig. 4) [14, 15]. The mechanism is often a direct blow to the ulna or a fall on an outstretched hand. Monteggia fracture-dislocations can lead to chronic radiocapitellar instability, nonunion/malunion of the ulna, and nerve complications. Open reduction and internal fixation of the ulna fracture and closed reduction of the radial head is the treatment of choice for Monteggia fracture-dislocations [16, 17].

### Essex-Lopresti fracture-dislocation

Presented by the young surgeon Peter Essex-Lopresti in 1951, the rare yet clinically significant Essex-Lopresti fracture-dislocation refers to the following constellation of findings: comminuted fracture of the radial head, dislocation of the DRUJ, and disruption of the interosseous membrane [18–20]. This injury requires a large amount of axial force on the forearm and elbow and usually occurs due to a fall on an outstretched hand. A radial head fracture should prompt dedicated radiographic imaging of the wrist to evaluate for DRUJ instability, similar to the Galeazzi fracture. Essex-Lopresti fractures usually require open reduction and internal fixation of the radial head and stabilization of the DRUJ through repair of the triangular fibrocartilage complex (TFCC). If the radial head comminution is severe, replacement of the radial head may be required [20, 21].

### Colles fracture (Pouteau)

**Fig. 5:**
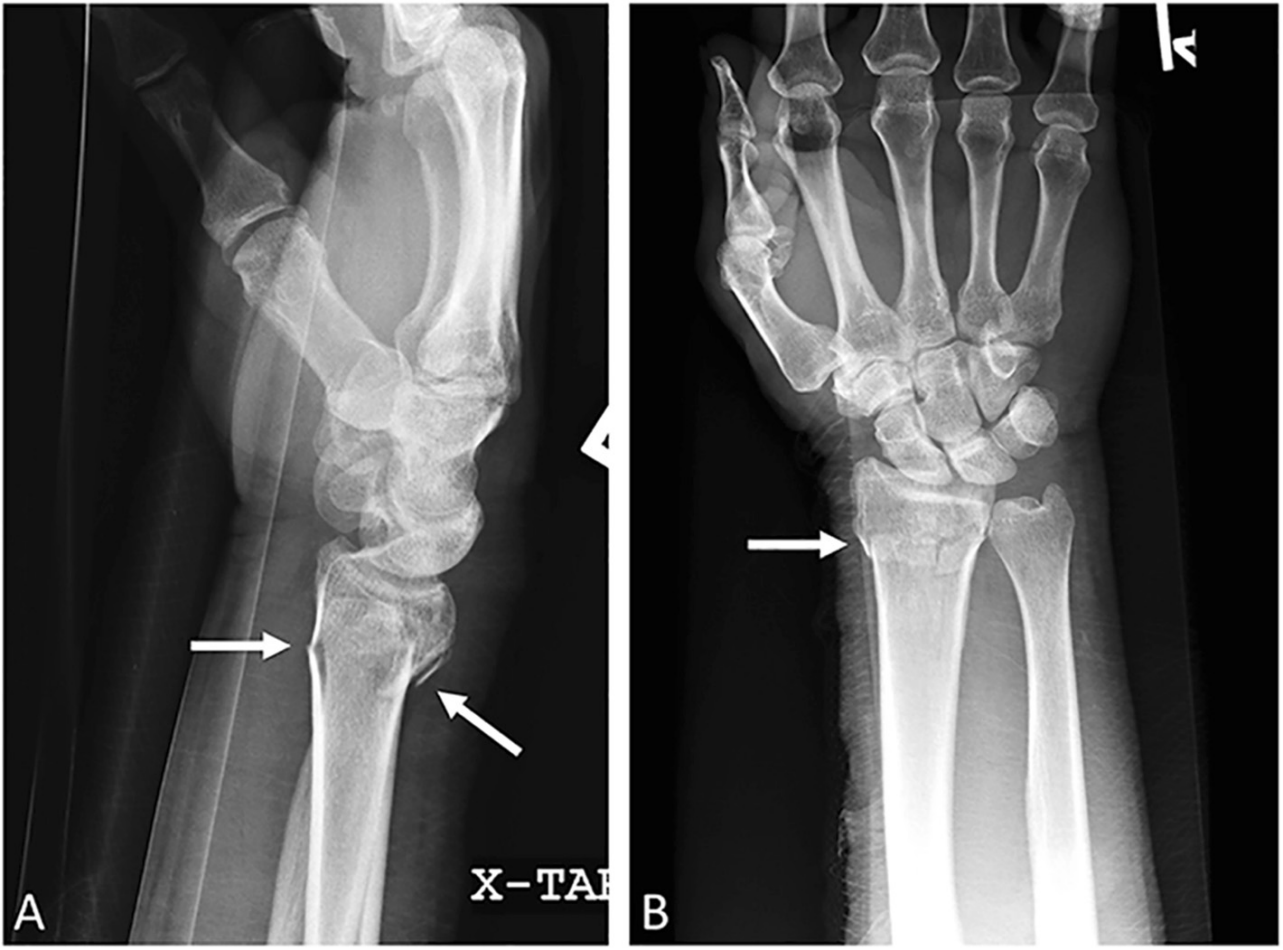
Colles fracture. Lateral (**a**) and AP (**b**) wrist radiographs. Transverse distal radial metaphyseal fracture (*arrows*) with no intra-articular extension. Mild dorsal angulation of the distal with respect to proximal fracture fragment; it is this angulation that differentiates the Colles fracture from the Smith fracture, which has volar angulation or displacement

An extremely common fracture seen in the emergency department, the Colles fracture, named after the Irish surgeon Abraham Colles, refers to an extra-articular transverse fracture of the distal radius with dorsal displacement of the distal radial fragment (Fig. 5) [22]. The clinical appearance of this fracture is known as the “dinner fork deformity” [23]. The mechanism of injury is a forward fall on an outstretched arm [22, 24, 25]. AP and lateral x-rays are often sufficient for diagnosis [10, 26]. An oblique view of the wrist can help in evaluation for fracture involvement of the articular surface and may aide in characterization of the DRUJ. Factors such as the degree of radial shortening, dorsal angulation, radial angulation, ulnar variance, and comminution contribute to fracture stability. Similar to the Smith fracture, treatment of a Colles fracture is nonsurgical, with closed reduction and splinting. An alternative name for the Colles fracture is the Pouteau fracture.

### Smith fracture (Goyrand, reverse Colles, reverse Bartons)

The Irish surgeon and pathologist Robert Smith originally described the Smith fracture as a transverse fracture of the distal radius with volar displacement and/or angulation of the distal fracture fragment. This fracture is usually the result of a fall on a flexed wrist [27]. With distal radius fractures, AP and lateral x-rays are sufficient for diagnosis and characterization. Associated findings may also include ulnar styloid fracture and possible subluxation or dislocation of the DRUJ [10, 22]. Treatment is usually nonsurgical with closed reduction and immobilization. More complicated cases with severe comminution may require open reduction and fixation [22]. Smith fractures may involve the articular surface, in which case it is also known as a volar-type Barton’s fracture (see “Barton’s fracture” section below). Other eponyms for this injury include the Goyrand fracture in French literature, reverse Colles fracture, or reverse Barton’s fracture.

### Barton’s fracture

**Fig. 6:**
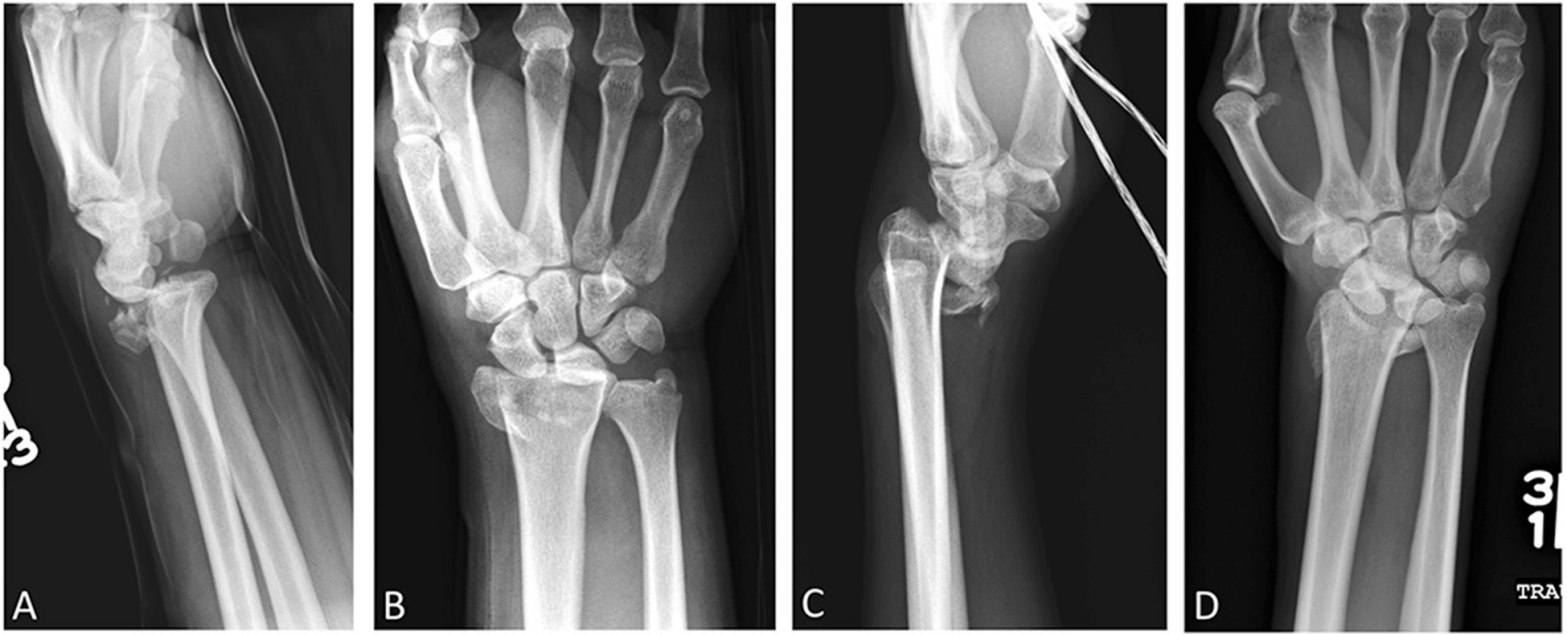
Barton’s and reverse Barton’s. Lateral (**a**) and AP (**b**) right wrist radiographs. Comminuted oblique intra-articular fracture with dorsal migration of the carpus, in keeping with Barton’s fracture. **c**, **d** Similar fracture morphology but located at the ventral aspect of the radius, with ventral carpal displacement

Not to be confused with the Colles and Smith fractures, the Barton’s fracture, first described by the American surgeon John Rhea Barton in 1838 [27], is an oblique fracture that extends to the articular surface of the distal radius (Fig. 6). The resultant triangular distal radial fragment is sheared off and displaced in the dorsal or volar direction along with the carpus [10, 27]. When the displacement is dorsal, this is known as a Barton’s fracture, whereas with volar displacement, this is termed a reverse Barton’s fracture. The mechanism of injury for the Barton’s and reverse Barton’s fracture is similar to the Colles’ fracture and Smith fracture, respectively. Barton fractures are unstable and can frequently result in osteoarthrosis, malunion, and instability; thus, surgical external fixation or open reduction and internal fixation is often required, particularly in reverse Barton’s fractures [28, 29].

### Thumb fractures

Injuries to the thumb are common as well as significant; an injury to the thumb can lead to significant loss of function of the hand. Two eponymous fractures involve the base of the first metacarpal, the Bennett and Rolando fractures, both of which are intra-articular fractures resulting from axial loading on the flexed metacarpal phalangeal joint.

#### Bennett fracture

**Fig. 7:**
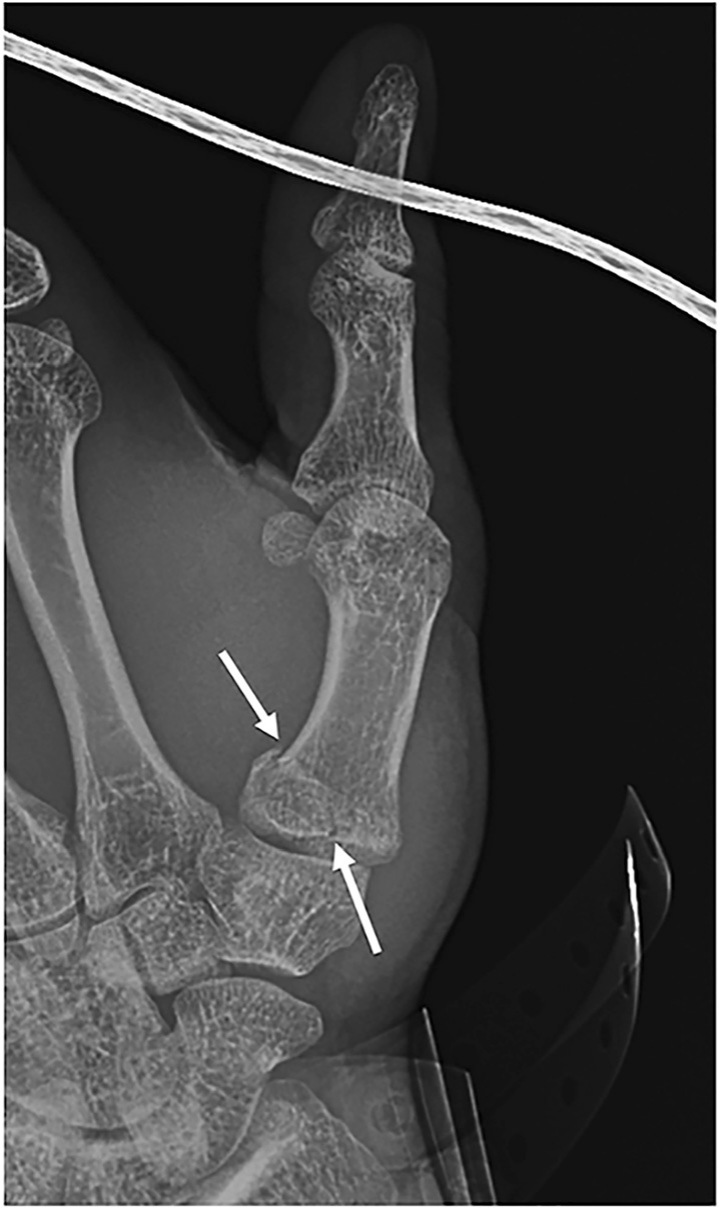
Bennett fracture. This single radiographic view of the thumb demonstrates an oblique intra-articular fracture at the base of the first metacarpal (*arrows*)

Edward Bennett, who succeeded Irish surgeon Robert Smith of the Smith fracture eponym as the Professor of Surgery at Trinity College, first described the Bennett fracture as an oblique intra-articular fracture at the base of the first metacarpal separating the volar-ulnar aspect of the metacarpal base from the remaining distal metacarpal shaft (Fig. 7) [30]. The volar-ulnar fragment is held in place via the volar anterior oblique ligament, while the large distal fragment is retracted proximally due to the tension from the abductor pollicis tendon [30, 31]. AP, lateral, and oblique views are typically obtained for diagnosis [30]. The degree of comminution, articular incongruity, and fracture fragment displacement are key components for treatment and prognosis. Complications include malunion, post-traumatic arthritis, and decreased range of motion [30]. If there is significant articular step off (>2 mm), then management is surgical through closed reduction with percutaneous pinning or open reduction with pins or interfragmentary fixation [30, 32]. Results are best achieved when residual displacement is less than 1 mm regardless of the method of treatment.

#### Rolando fracture

**Fig. 8:**
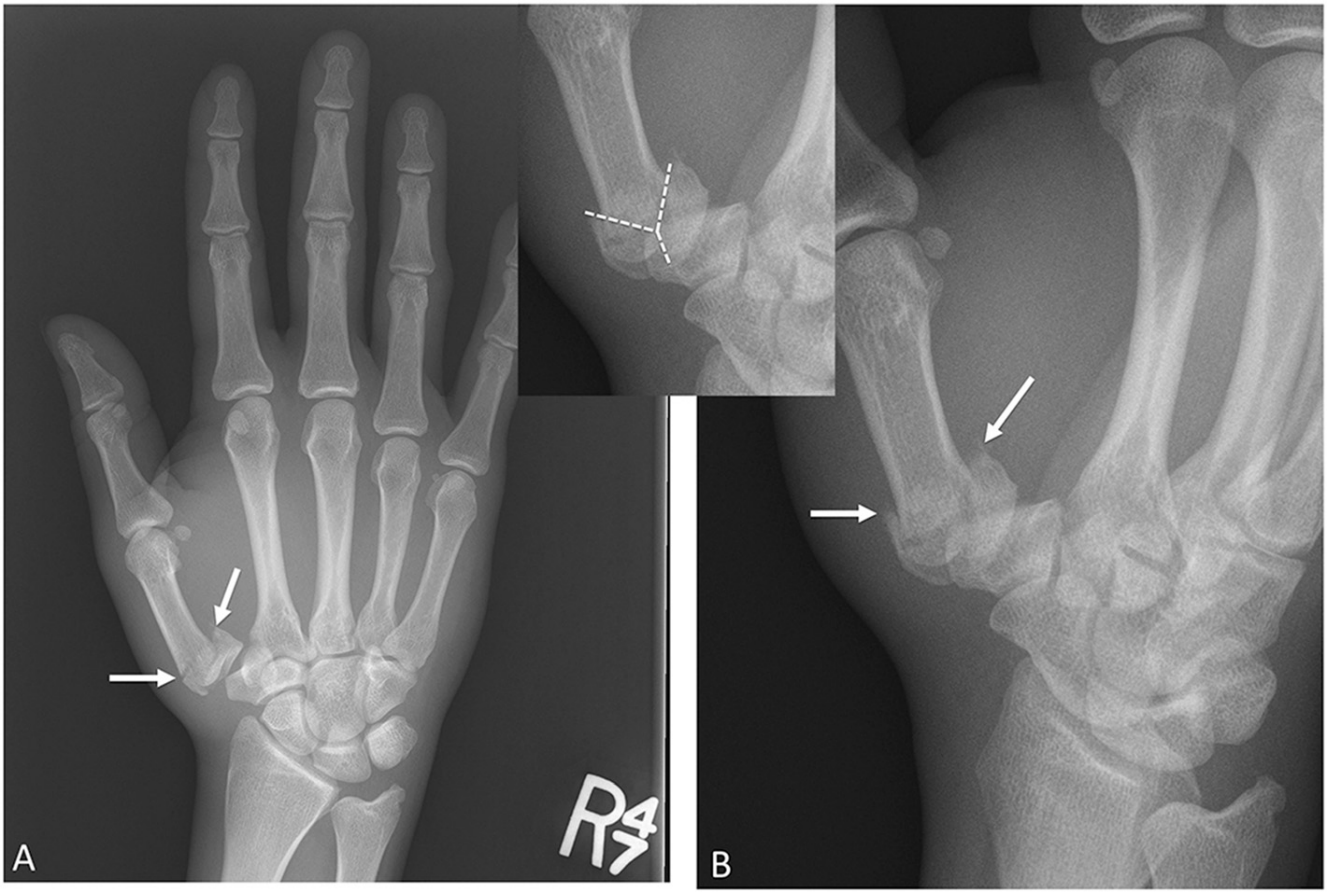
Rolando fracture. AP (**a**) and oblique radiographs (**b**). Comminuted fracture at the base of the first metacarpal (*arrows*), with intra-articular extension into the first carpal-metacarpal joint. *Inset* picture delineating the typical “Y” shaped fracture pattern. These fracture patterns can also be “T” shaped

Although less common than the Bennett fracture, the Rolando fracture is a more severe fracture of the metacarpal base. It was originally described by Silvio Rolando as a comminuted, intra-articular fracture at the base of the first metacarpal with three major segments: the metacarpal shaft, the volar fragment, and the dorsal fragment, creating a T- or Y-shaped fracture pattern (Fig. 8) [33, 34]. The Rolando fracture is now known as an intra-articular thumb metacarpal base fracture with three or more segments. Complications are similar to those of the Bennett fracture. The comminution results in instability and requires surgical treatment, which is often difficult due to the fracture fragments [30, 32].

## Conclusions

Fracture eponyms of the extremities are frequently used in everyday practice by radiologists, emergency clinicians, and orthopedists. Accurate knowledge of eponymous fractures can facilitate patient care by helping radiologists and emergency clinicians efficiently convey a great deal of information in an extremely concise manner. For a brief summary of the reviewed upper extremity fracture eponyms, please see Table 1. The second manuscript of this two-part series will review fracture eponyms of the lower extremities.

**Table 1:** Upper extremity fracture eponyms

Upper extremity fracture eponyms	Fracture pattern
Hill-Sachs	Impaction fracture of the posterolateral aspect of the humeral head
Bankart	Chip fracture of the anterior inferior glenoid cortical rim (bony Bankart).
Holstein-Lewis	Spiral fracture of the distal third of the humerus. Twenty-two percent are associated with radial nerve palsy
Galeazzi	Fracture of the middle to distal third of the radius and associated dislocation of the distal radioulnar joint
Monteggia	Fracture of the proximal ulna with dislocation of the proximal radioulnar and radiocapitellar joints
Essex-Lopresti	Comminuted fracture of the radial head, dislocation of the distal radioulnar joint, and disruption of the interosseous membrane
Colles	Extra-articular transverse fracture of the distal radius with dorsal displacement of the distal radius fragment
Smith	Transverse fracture of the distal radius with volar displacement and/or angulation of the distal fracture fragment
Barton	Oblique intra-articular fracture of the distal radius with resultant volar or dorsal displacement of the distal fragment. The radiocarpal joint is intact.
Bennett	Oblique intra-articular fracture at the base of the first metacarpal
Rolando	Comminuted, intra-articular fracture at the base of the first metacarpal, typically with three major fragments, creating a T- or Y-shaped fracture pattern

## Abbreviations

AP: anterior-posterior
CT: computed tomography
DRUJ: distal radioulnar joint
MRI: magnetic resonance imaging
TFCC: triangular fibrocartilage complex

## References

[1] Woywodt A, Matteson E (2007). Should eponyms be abandoned? Yes. BMJ.

[2] Gyftopoulos S, Albert M, Recht MP (2014). Osseous injuries associated with anterior shoulder instability: what the radiologist should know. AJR Am J Roentgenol.

[3] Harris JH, Thomas P (2013). The radiology of emergency medicine.

[4] Sheehan SE, Gaviola G, Gordon R, Sacks A, Shi LL, Smith SE (2013). Traumatic shoulder injuries: a force mechanism analysis-glenohumeral dislocation and instability. AJR Am J Roentgenol.

[5] Holstein A, Lewis GM (1963). Fractures of the humerus with radial-nerve paralysis. J Bone Joint Surg Am.

[6] Ekholm R, Ponzer S, Tornkvist H, Adami J, Tidermark J (2008). The Holstein-Lewis humeral shaft fracture: aspects of radial nerve injury, primary treatment, and outcome. J Orthop Trauma.

[7] Shao YC, Harwood P, Grotz MR, Limb D, Giannoudis PV (2005). Radial nerve palsy associated with fractures of the shaft of the humerus: a systematic review. J Bone Joint Surg (Br).

[8] Carlsen BT, Dennison DG, Moran SL (2010). Acute dislocations of the distal radioulnar joint and distal ulna fractures. Hand Clin.

[9] Squires JH, England E, Mehta K, Wissman RD (2014). The role of imaging in diagnosing diseases of the distal radioulnar joint, triangular fibrocartilage complex, and distal ulna. AJR Am J Roentgenol.

[10] Porrino JA, Maloney E, Scherer K, Mulcahy H, Ha AS, Allan C, Mulcahy H, Ha AS, Allan C (2014). Fracture of the distal radius: epidemiology and premanagement radiographic characterization. AJR Am J Roentgenol.

[11] Nakamura R, Horii E, Imaeda T, Tsunoda K, Nakao E (1995). Distal radioulnar joint subluxation and dislocation diagnosed by standard roentgenography. Skeletal Radiol.

[12] Giannoulis FS, Sotereanos DG (2007). Galeazzi fractures and dislocations. Hand Clin.

[13] Lee P, Hunter TB, Taljanovic M (2004). Musculoskeletal colloquialisms: how did we come up with these names?. Radiographics.

[14] Bado JL (1967). The Monteggia lesion. Clin Orthop Relat Res.

[15] Ring D (2013). Monteggia fractures. Orthop Clin North Am.

[16] Rehim SA, Maynard MA, Sebastin SJ, Chung KC (2014). Monteggia fracture dislocations: a historical review. J Hand Surg [Am].

[17] Eathiraju S, Mudgal CS, Jupiter JB (2007). Monteggia fracture-dislocations. Hand Clin.

[18] Essex-Lopresti P (1951). Fractures of the radial head with distal radio-ulnar dislocation; report of two cases. J Bone Joint Surg (Br).

[19] Dodds SD, Yeh PC, Slade JF (2008). Essex-Lopresti injuries. Hand Clin.

[20] Wegmann K (2012). The Essex-Lopresti lesion. Strategies in Trauma and Limb Reconstruction.

[21] McGlinn EP, Sebastin SJ, Chung KC (2013). A historical perspective on the Essex-Lopresti injury. J Hand Surg [Am].

[22] Goldfarb CA, Yin Y, Gilula LA, Fisher AJ, Boyer MI (2001). Wrist fractures: what the clinician wants to know. Radiology.

[23] Roche CJ, O’Keeffe DP, Lee WK, Duddalwar VA, Torreggiani WC, Curtis JM (2002). Selections from the buffet of food signs in radiology. Radiographics.

[24] Loredo RA, Sorge DG, Garcia G (2005). Radiographic evaluation of the wrist: a vanishing art. Semin Roentgenol.

[25] Flemming C (1933). Colles’s fracture. Postgrad Med J.

[26] Breitenseher MJ, Gaebler C (1997). Trauma of the wrist. Eur J Radiol.

[27] Ellis J (1965). Smith’s and Barton’s fractures. A method of treatment. J Bone Joint Surg (Br).

[28] Aggarwal AK, Nagi ON (2004). Open reduction and internal fixation of volar Barton’s fractures: a prospective study. J Orthop Surg (Hong Kong).

[29] Pattee GA, Thompson GH (1988). Anterior and posterior marginal fracture-dislocations of the distal radius. An analysis of the results of treatment. Clin Orthop Relat Res.

[30] Carlsen BT, Moran SL (2009). Thumb trauma: Bennett fractures, Rolando fractures, and ulnar collateral ligament injuries. J Hand Surg [Am].

[31] Brownlie C, Anderson D (2011). Bennett fracture dislocation—review and management. Aust Fam Physician.

[32] Soyer AD (1999). Fractures of the base of the first metacarpal: current treatment options. J Am Acad Orthop Surg.

[33] Rolando S (2006). Fracture of the base of the first metacarpal and a variation that has not yet been described: 1910. (Translated by Roy A. Meals). Clin Orthop Relat Res.

[34] William Brant CH (2012). Fundamentals of diagnostic radiology.

